# Robust health-score based survival prediction for a neonatal mouse model of polymicrobial sepsis

**DOI:** 10.1371/journal.pone.0218714

**Published:** 2019-06-24

**Authors:** Byron Brook, Danny Harbeson, Nelly Amenyogbe, Rym Ben-Othman, Tobias R. Kollmann, Radhouane Aniba

**Affiliations:** 1 Department of Experimental Medicine, University of British Columbia, Vancouver, BC, Canada; 2 Department of Pediatrics, Division of Infectious Diseases, University of British Columbia, Vancouver, BC, Canada; 3 Department of Molecular Oncology, BC Cancer Agency, Vancouver, BC, Canada; 4 Department of Pathology and Laboratory Medicine, University of British Columbia, Vancouver, BC, Canada; Biotechnology HPC Software Applications Institute (BHSAI), UNITED STATES

## Abstract

Infectious disease and sepsis represent a serious problem for all, but especially in early life. Much of the increase in morbidity and mortality due to infection in early life is presumed to relate to fundamental differences between neonatal and adult immunity. Mechanistic insight into the way newborns’ immune systems handle infectious threats is lacking; as a result, there has only been limited success in providing effective immunomodulatory interventions to reduce infectious mortality. Given the complexity of the host-pathogen interactions, neonatal mouse models can offer potential avenues providing valuable data. However, the small size of neonatal mice hampers the ability to collect biological samples without sacrificing the animals. Further, the lack of a standardized metric to quantify newborn mouse health increases reliance on correlative biomarkers without a known relationship to ‘clinical’ outcome. To address this bottleneck, we developed a system that allows assessment of neonatal mouse health in a readily standardized and quantifiable manner. The resulting health scores require no special equipment or sample collection and can be assigned in less than 20 seconds. Importantly, the health scores are highly predictive of survival. A classifier built on our health score revealed a positive relationship between reduced bacterial load and survival, demonstrating how this scoring system can be used to bridge the gap between assumed relevance of biomarkers and the clinical outcome of interest. Adoption of this scoring system will not only provide a robust metric to assess health of newborn mice but will also allow for objective, prospective studies of infectious disease and possible interventions in early life.

## Introduction

An estimated 3.0 million cases of newborn infection every year result in nearly 350,000 deaths, making infection one of the most lethal threats in early life [1]. Many deaths due to infection can be attributed to sepsis, a difficult to define disease characterized by failures of regulatory immune system components resulting in hyper- or hypo-inflammation [2,3]. Sepsis may be caused by an invasive bacteria or virus, leakage of commensal microbes from the gut, exposure to maternal vaginal flora or any number of inflammatory stimuli[4,5]. It is widely known that the neonatal immune system is fundamentally distinct from that of adults, indicating that data generated from adult animal models should not be assumed to apply to newborns [6–9]. While neonatal mouse models offer several practical benefits (availability, existing infrastructure, well-understood biology, etc.), working with neonatal mice presents some unique challenges which have not yet been effectively addressed. In particular, researchers are limited by the size of the mice; day of life (DOL) 7 mice (considered to be immunologically closest to human term newborns at birth) [10] are still far too small to collect meaningful volumes of blood without sacrificing experimental animals. In adult mice, about 7 to 8 μL of blood can ethically be collected per gram of mouse bodyweight without sacrifice or fluid replacement–this translates to a maximum single collectible blood volume from neonatal mice of about 20 μL [11]. Serial sampling (every 24 hours) would only allow for collection of 5 μL of blood or less in each draw [12]. This limitation (in addition to the practical difficulties of drawing blood from such small animals) only allows terminal methods of bleeds to collect samples of adequate volume, which fundamentally separates biological findings from true ‘clinical’ outcomes (i.e. survival). This inability to serially sample neonatal mice without sacrifice also forces researchers to rely heavily on biomarkers as a stand-in for outcome when exploring a potential treatment of interest, assuming causality between e.g. pathogen load or inflammatory cytokine production and mortality.

Health scores for sepsis are widely used in human clinical settings as they are critical for capturing disease progression and can inform potential interventions [13–17]. The ability to assess disease severity in a quantitative way is also extremely useful in animal models as it can help investigators avoid an overreliance on assumptions about the relevance of a given biomarker. For example, one group developed a series of symptom scores for adult mice in a similar model of sepsis (fecal suspension injected IP) which relied on, among other things: activity, response to stimulus, eyes (open or shut), level of consciousness, and appearance [18]. This study illustrated that when grouping adult mice by these health scores, TNFα and IL-1β levels did not correlate with outcome despite elevated levels in comparison to unchallenged controls–these cytokines went up following challenge but did not appear different in survivors and non-survivors. This demonstrates the capacity for health scores to narrow the focus of investigators onto biomarkers which are functionally relevant to a clinical outcome of interest–a capacity even more critical when working in a neonatal population from which even blood samples are extremely difficult to collect without harming them. While the quantitative observations used in this model may be strong markers for adult animals, none of these characteristics are readily applicable to a neonatal population (i.e. no fur to be ruffled, eyes are always closed, minimal basal activity levels, etc.).

To address this need, we developed a data-driven and simple scoring system directly related to outcome (humane endpoint) in neonatal mice. The addition of our health scores to neonatal mouse experiments provides: (a) an improved ability to track disease progression which can inform time of sacrifice for sample collection and (b) a quantitative method to prospectively differentiate survivors from non-survivors. We here correlate this health scoring system with mortality in a polymicrobial model of neonatal sepsis. This model confirms that clinical outcome was highly correlated with bacterial load. It took less than 20 seconds to determine these composite health scores per animal. The prospective validity of our health score in assigning outcome provides the much-needed metric to begin assessing causality of biomarkers, which in turn will allow assignment of mechanistic cause-effect relationships. This approach will not only decrease undue suffering of the animals, but also increases the likelihood of identifying effective immunomodulatory interventions for infectious disease and sepsis.

## Materials and methods

### Mice

Male and female C57BL/6 mice aged 6 weeks (Jackson Labs, catalogue #664) were purchased from The Jackson Laboratory and used for breeding purposes; all mice used in this study were first or second-generation progeny of Jackson mice bred in-house. This study was carried out in strict accordance with animal ethics guidelines outlined by University of British Columbia and was approved by the Animal Care Committee (protocol numbers A17-0110 and A14-0261). Animals were housed in OptiMice cages, with food and water *ad libitum*. Breeding mice were fed a high fat diet (Tekland Cat 2919) while others were fed a low-fat diet (Tekland Cat 2918). The pellet base was cellulose ¼” Performance Bedding (Lab Animal Supplies INC, Cat L0108), and environmental enrichment included a red hut, nesting material of Envirodri (Sic.) (Jameson, B501) and Nestlets (Ancare, Cat CABFM00088). Mice underwent a controlled 12 hour light cycle, with temperature maintained at 23°C Mice were anesthetized with isoflurane prior to euthanasia through CO_2_ exposure, confirmed by decapitation. Humane endpoint was defined as the lack of an attempt to right themselves when placed on their back on both sides, explained in greater detail below.

### Cecal slurry model of neonatal sepsis

Polymicrobial sepsis was induced using a modified version of the model first described by Wynn *et al*. in 2007 and recently outlined on video in the Journal of Visualized Experiments [19,20]. Briefly, cecal material was extruded from adult male mouse ceca (aged 6–10 weeks), diluted in dextrose 5% water (D5W) to 160 mg slurry / mL D5W, filtered through a 70 μm sterile strainer, pooled, aliquoted, and frozen at -80 ^o^C. Aliquots were thawed at room temperature, diluted further in D5W to the desired weight-adjusted dose, and kept on ice prior to intraperitoneal (IP) injection. The challenge dose was titrated to achieve a lethal dose equal to 50% (LD50) per litter. Neonatal mice were injected IP with approximately 100 μL cecal slurry (0.8–1.1 mg/g mouse) on day of life (DOL) 7 or 8 to induce polymicrobial sepsis, with slight variations in volume due to weight adjustment. Litters were monitored for morbidity (i.e. lethargy, inability to right) up to 96 hours post challenge (HPC). In these survival experiments, mice were monitored either until full recovery was observed (gaining weight, alert / active), or until humane endpoint was reached at which point the mice would be euthanized. A separate cohort of pups were sacrificed at approximately 24 HPC in order for the collection of blood and tissue samples. Bacterial loads in liver, lungs, spleen, and blood were subsequently calculated by serially diluting the organ homogenates in PBS, drop-plating on 5% sheep’s blood agar, and the counting colony forming units (CFU) after 24 hours of incubation at 37 ^o^C.

### Monitoring and health scores

Monitoring commenced 12 HPC (i.e. prior to the onset of expected mortality) and continued 2–3 times daily (07:00–09:00, 12:00–14:00, 16:00–18:00, spanning both light and dark cycles) through to experimental endpoint. All mice were challenged in the evening (17:00–18:00) so monitoring schedules were not adjusted based on time of challenge. During mouse scoring the neonatal mice were separated into a secondary cage to avoid stressing the dam. Nesting material was rubbed in hands to transfer that litter’s smell to the gloves, and then each mouse was individually scruffed and placed on their back as visualized in Brook *et al*. 2019 [20]. Health scores arose naturally out of a need to define a humane endpoint for extremely ill neonatal pups. Placing a pup on its back and recording whether it was able to right itself was a simple and effective discriminator of outcome, but alone it failed to capture the obvious difference between mice which were vigorously attempting to right and those which barely moved. Thus, we began recording qualitative observations of vigor to pair with righting reflex data, which eventually became quantitative descriptors of mobility (i.e. angle of hip movement when attempting to right). Righting reflex measurements were further standardized by adding a four-second cutoff, determined by the observation that more time was unnecessary, and less time risked inappropriately assigning ‘fail to right’ to a healthy pup. Previous work has shown minimal variance in score assignments between trained individuals[20]. For simplicity of recording and interpretation, these observations were transformed into ranked scores from 0–5, as presented in Table 1. Scores were recorded at each monitoring time point twice, once with the mouse placed on its back-right and once on the back-left side. In addition to scoring, mice were weighed at each time point using a top-loading balance. All mice used were either 7 or 8 days old and weighed an average of 3.7 g (2.25 g—6.06 g). Pups were identified by markings with an ethanol proof pen on the top or bottom of the tail. Monitoring sheets for each litter were used to record scores and weights. A total of 424 mice were used in this study: 266 with an endpoint of mortality or recovery, 125 sacrificed at 24 hours for a readout of bacterial load, 33 used in follow-up experiments. In survival experiments, all mice were allowed to proceed to humane endpoint or recovery (gaining weight four days post-challenge with scores of 4–5), whereas in bacterial load experiments all mice were sacrificed at 24 HPC regardless of perceived illness.

**Table 1:** Assigning numerical health scores to quantitative observations in neonatal mice.

Numerical Score	Righting Reflex*	Mobility	Description
5	Rights	Mobile	• Multiple steps in a row, responsive to tail tap • Alert, may be shaky but moving with energy • Might fall over while walking
4	Rights	Lethargic	• Takes at least one step, but stops between steps • Steps are slower, may be very shaky, may fall over
3	Rights	Nonmobile	• Stationary after righting • May take a half-step after tail tap but largely or entirely unresponsive
2	Fails to right	Mobile	• Vigorous hip movement, swinging side to side at an angle > 90° from horizontal at least once during the observation period (4 seconds)
1	Fails to right	Lethargic	• Slower hip movement, < 90° angle • Halting attempts to right over the observation period, may be some pauses but always starting again
0	Fails to right	Nonmobile	• Zero or extremely weak hip movement • Gives up attempting to right or never starts, may be uncontrollably shaky • limbs may bend at the knee and extend but hips will not rock side to side

*Righting reflex measured by placing mice gently on their back and observing their ability to right within 4 seconds. All scores were taken in duplicate with the lower of the two reported here.

### Classifier

The ability to confidently predict outcome (survival or non-survival) at time of sacrifice would greatly help limit the number of assumptions required by investigators. We interpreted survival vs. non-survival as a binary classification problem and constructed multiple models aimed at classifying mice into one of these two groups at 24 HPC. In order to identify the best model, we tested our dataset in 6 different algorithms split across two primary classes: baseline learning algorithms / simple regression (Logistic Regression, K Nearest Neighbors and Decision Tree) and ensemble learning algorithms (Random Forest, Gradient Boosting and XGBoost). Feature selection began from all available data collected for each mouse: sex, date of birth, time of challenge, weight at challenge, weight at each monitoring time point, precise HPC each monitoring time occurred, righting reflex, mobility at each monitoring time point, and the change in any of the above from one point to another. Pearson correlation coefficients were used to avoid including highly co-correlated and irrelevant features, with a correlation threshold of 0.2 (with outcome) acting as the minimum cutoff for inclusion. This threshold and all other parameters were optimized iteratively using GridSearch, which examines all possible combinations of values for each parameter and tunes each parameter to maximize classification strength. A more detailed description of the other approaches we examined for feature selection is presented in the supplementary material (S1 Appendix). For specific parameters used during classifier construction, see the source code linked in the supplementary materials (S1 URL). The righting reflex and mobility were treated separately (rather than combined as scores) to maximize the amount of information available for the classification problem and minimize the potential for biasing what should be an unsupervised process with our own assumptions about what should be associated with mortality. A total of 222 mice were split into a training set (148 mice) and a test set (74 mice) and the different approaches were evaluated by accuracy (total number of correct classifications over total number of instances) and area under the receiver operating characteristic curve (AUC), which quantified sensitivity and specificity and represented a good metric of the quality of a classifier (S1 Table). Evaluation based on these performance metrics led us to select Gradient Boosted Machine learning as the strongest classification algorithm and was applied to an external data set of 21 mice for further validation. A detailed description of classifier construction and background behind the various approaches examined are available in the supplementary material (S1 Appendix).

### Statistics

Comparisons of survival curves were evaluated using the log-rank test, with statistical significance assigned when *p* < 0.05. All tests comparing score and survival excluded the assigned score of 0, as this was the defined humane endpoint and thus could not be treated as independent. Percent mortality was analyzed using two-sided Fischer’s exact tests with Bonferroni adjustment where appropriate. Scores were only treated as numerical quantities for the purpose of examining change over time–all other analyses treated scores as categorical. Any statistical test applied identically to blood, spleen, liver, and lung was adjusted using the Bonferroni method. Bacterial load data did not exhibit a normal distribution (per Shapiro-Wilk normality test) and were compared using the Wilcoxon rank-sum test. When analyzing independent variables with multiple levels, data were first tested with the non-parametric Kruskal-Wallis test and only if significant (*p* < 0.05) were post-hoc, pairwise comparisons made using the Wilcoxon rank-sum test (Benjamani-Hochberg adjusted). All statistical analyses were performed in R (version 3.5.2).

## Results

### Health scores and outcome

**Fig 1:**
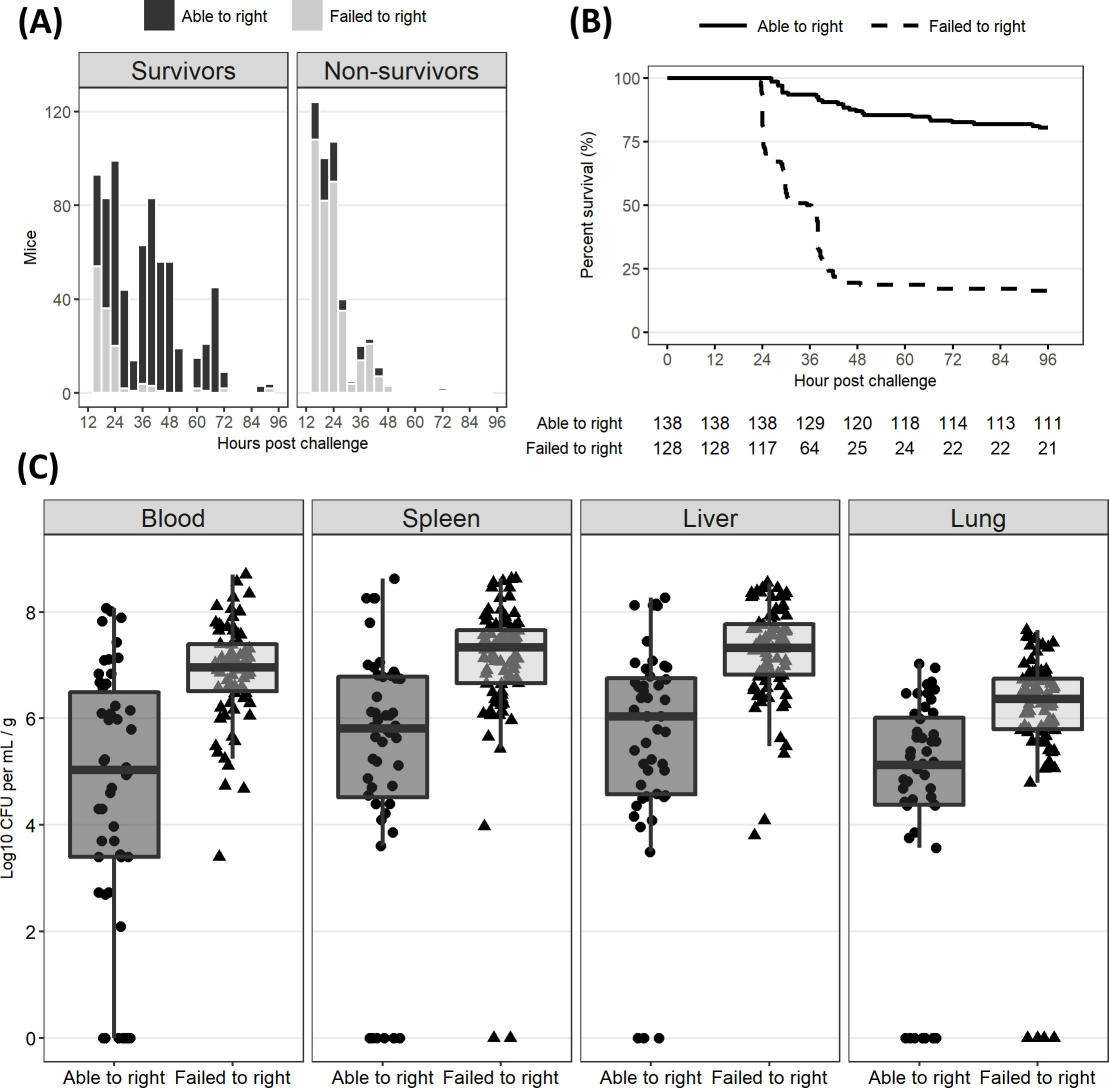
Righting reflex at 24 HPC is an excellent discriminator of survival and bacterial load. Righting reflex of neonatal mice (DOL 7–8) challenged IP with cecal slurry at an LD50. (A) Ability to right at different hours post challenge (HPC) in survivors and non-survivors. (B) Survival curve of mice split by righting reflex at 24 HPC. Mice which were able to right themselves at 24 hours were significantly more likely to survive the cecal slurry challenge than mice which failed to right (log-rank test, p < 0.001). (C) Bacterial load split by righting reflex at time of sacrifice, 24 HPC. Mice which were able to right had significantly lower bacterial load in blood and all measured organ tissues (two-sided Wilcoxon rank-sum tests with Bonferroni correction, p < 0.001).

At all monitoring time points, righting was associated with survival and failure to right (FTR) was associated with non-survival (Fig 1A). As 24 HPC was as far into disease progression as one could wait to sacrifice mice without incurring heavy survivor bias (Fig 1B), this timepoint became the time of sacrifice for the alternative cohort of mice assessed for bacterial load. It was therefore critical to be able to distinguish survivors and non-survivors prior to sacrifice and no later than 24 HPC. Mice which failed to right at 24 HPC were significantly more likely to die than those which successfully righted (log-rank test, p < 0.001) and had a statistically significant, 100-fold higher bacterial load across all compartments (two-sided Wilcoxon rank-sum tests with Bonferroni correction, p < 0.001) (Fig 1C). Righting reflex alone was strongly correlated with survival and bacterial load, especially after 24 HPC. We also captured three different levels of mobility: non-mobile, lethargic, and mobile (Table 1). One of the strengths of a scoring system should be the ability to provide a metric predictive of future outcome. To assess the predictive value of this righting / mobility combination metric, we assigned scores from 0 (fail to right non-mobile, humane endpoint) to 5 (rights mobile, perfectly healthy) (Table 1). Unless otherwise indicated, the reported score is the lower of the two recorded for each mouse.

### Health scores are strongly associated with survival and bacterial load

**Fig 2:**
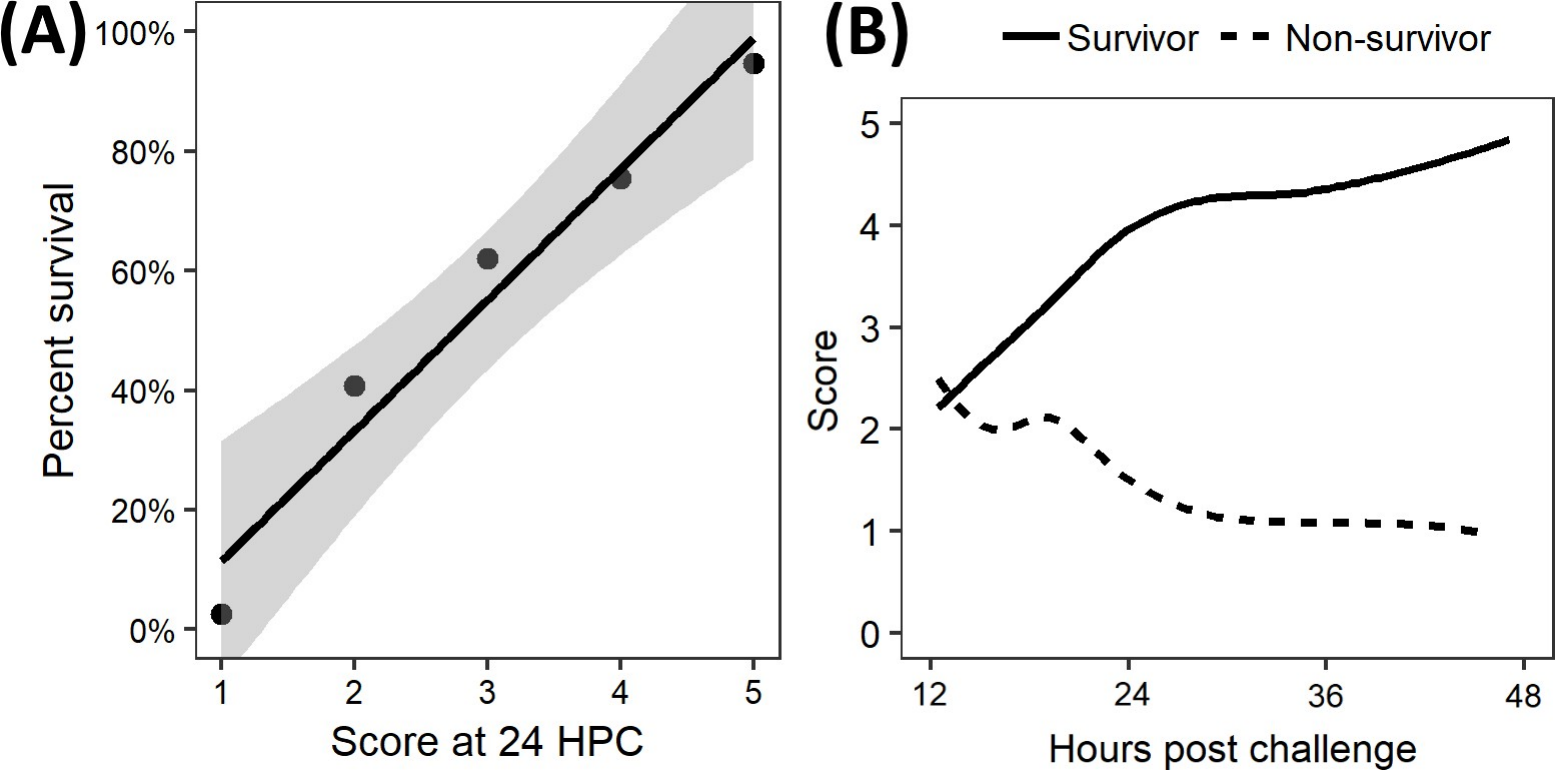
Health scores were directly associated with outcome in a polymicrobial model of sepsis in neonatal mice. (A) Scores recorded at 24 hours post challenge (HPC) plotted against the percent of mice which survived the IP cecal slurry challenge. Scores of zero were excluded as they were the pre-determined humane endpoint. A significant linear regression was found (R^2^ = 0.946, p = 0.0035, n = 210) with mean percent survival increasing by 22 ± 8% for each single point increase in score. Shaded area represents standard error. (B) Visual representation of change in score defined numerically over time following IP cecal slurry challenge.

Combining mobility with righting reflex allowed us to generate six categories which were assigned to numerical scores for ease of recording and visualizing. As the score of zero was not independent from mortality (score of zero was the defined humane endpoint and necessitated euthanasia), it was excluded from statistical analyses. Mice which received sham challenges with either phosphate buffered saline (PBS) or dextrose 5% water (D5W) exhibited no mortality and had significantly higher scores than those which received cecal slurry, indicating that the drop in score post-challenge was not a reflection of injury due to the injection itself but rather was in response to disease (S1 Fig). Comparing the categorical scores at 18 and 24 HPC indicated a significant relationship between score and survival (Fisher’s exact test, p < 0.001). A simple linear regression model examining percent overall survival against assigned score at 24 HPC (mice with a score of 0 were excluded) was found to be significant (R^2^ = 0.946, p = 0.0035, n = 210) with each increase in score equating to a 22% ± 8% improved chance of survival (Fig 2A). Visualizing the changes in score over the first 48 hours of disease (capturing 93% of deaths) showed a clear separation between survivors and non-survivors beginning around 18 HPC, with higher scores associated with recovery and survival (Fig 2B).

The inability to distinguish survivors through health scores at 12 HPC (Fig 2B) was consistent with qualitative observations that most mice appeared similar during this time period irrespective of final outcome. At 24 HPC, the scores were well distributed with the least frequent score (3) assigned to 10.9% of mice (29/266) and the most frequent, the healthiest score of 5, assigned to 21.0% (56/266) of mice (S2 Fig). At 18 HPC, many of the mice appeared similarly ill, with only 6.9% at the healthiest score of 5 and 46.8% at the middle score of 2. Thus, health scores at 24 HPC were most useful in their ability to capture a range of sickness in neonatal mice.

**Fig 3:**
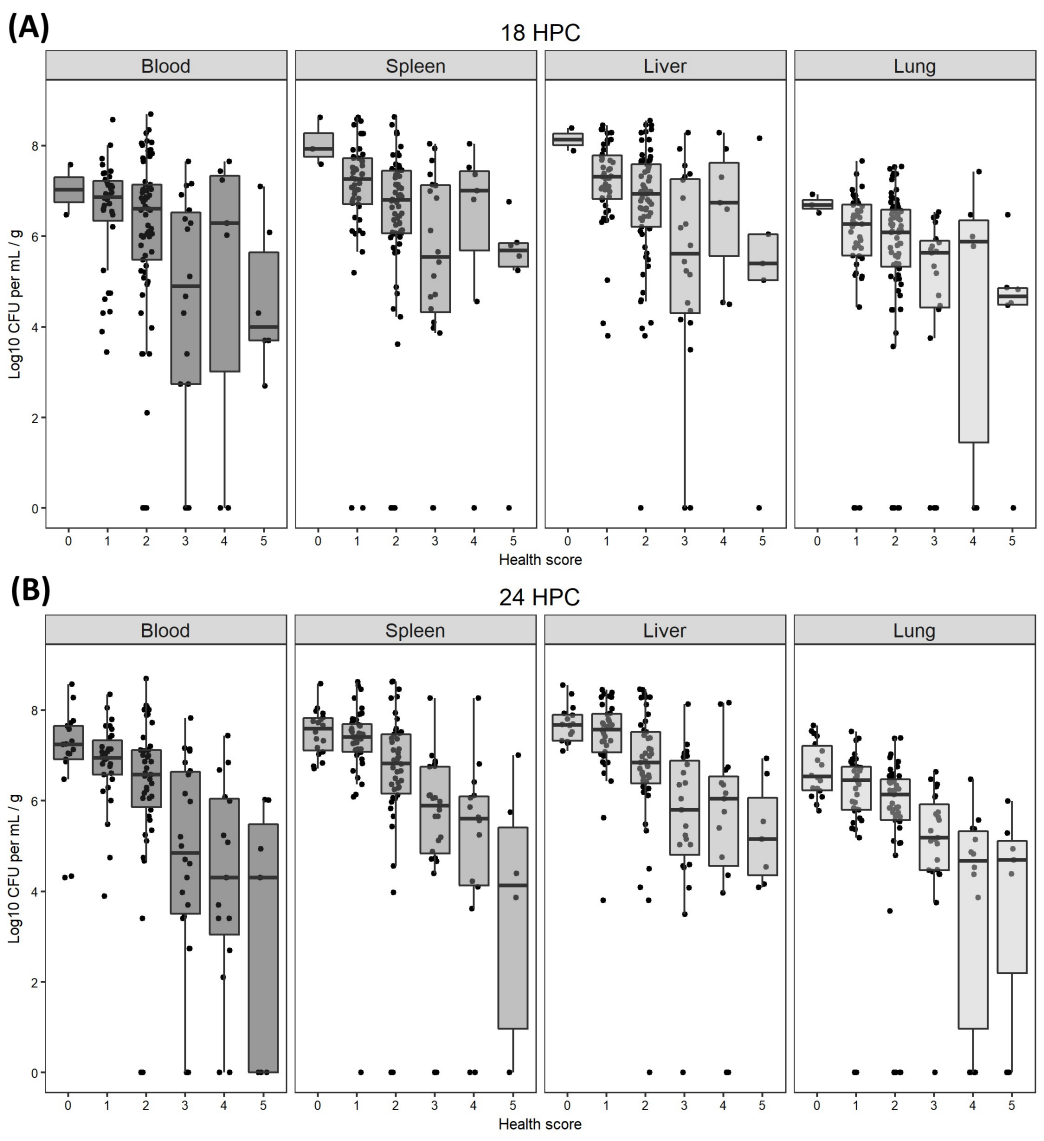
Health scores at 18 and 24 HPC are related to bacterial load at 24 HPC. (A) A Kruskal-Wallis comparison of score 18 hours post challenge (HPC) with bacterial load in whole-blood and tissues showed a significant effect (Bonferroni-adjusted p-values < 0.001) indicating score at 18 HPC was related to the bacterial load measured in tissues of the same animals collected 6 hours later (24 HPC). (B) A significant effect was also attained at 24 HPC (Bonferroni-adjusted p-values < 0.001) demonstrating the relationship between health scores and bacterial load across all biological compartments.

To test the validity of our scoring system, a separate cohort of 125 mice generated under the same experimental conditions were sacrificed at 24 HPC and samples of whole-blood, spleen, liver, and lung tissue were plated on sheep’s blood agar and the colony forming units (CFU) growth was assessed 24 hours later. Two separate, non-parametric Kruskal-Wallis tests were performed to assess the relationship between categorical scores at 18 and 24 HPC and bacterial load (at 24 HPC) across all four biological samples. A significant effect was attained both at 18 HPC (Bonferroni-adjusted p-values < 0.001) and 24 HPC (Bonferroni-adjusted p-values < 0.001) (Fig 3). The distribution of scores broadened as disease progressed towards an outcome–nearly all mice scored between 1–3 at 12 HPC, most fell within that same range at 18 HPC, but an even distribution was present at 24 HPC (S2 Fig). Our health scores were strongly associated with both survival and bacterial load at 24 HPC.

### Righting reflex and mobility are predictive of outcome prior to sacrifice

We used a machine learning approach to classify mice as survivors or non-survivors based primarily on righting reflex, mobility, and weight, specifically looking at how each metric changed from 18 to 24 HPC. Only 12% (28 / 222) of mice were at humane endpoint at 24 HPC, so while the association between score components and outcome may be influenced by the inclusion of these mice (mice which were FTR non-mobile on both sides are at humane endpoint and must be sacrificed), they did not represent a large enough proportion to call the results into question. Pearson correlation proved the most effective method of feature selection and identified attributes most associated with death: a mobility score of “non-mobile”, a change from able to right at 18 HPC to failed to right at 24 HPC, and weight loss between 18 and 24 HPC (S4 Fig). The ability to right was strongly correlated with survival, especially the consistent ability to right at both 18 and 24 HPC.

**Fig 4:**
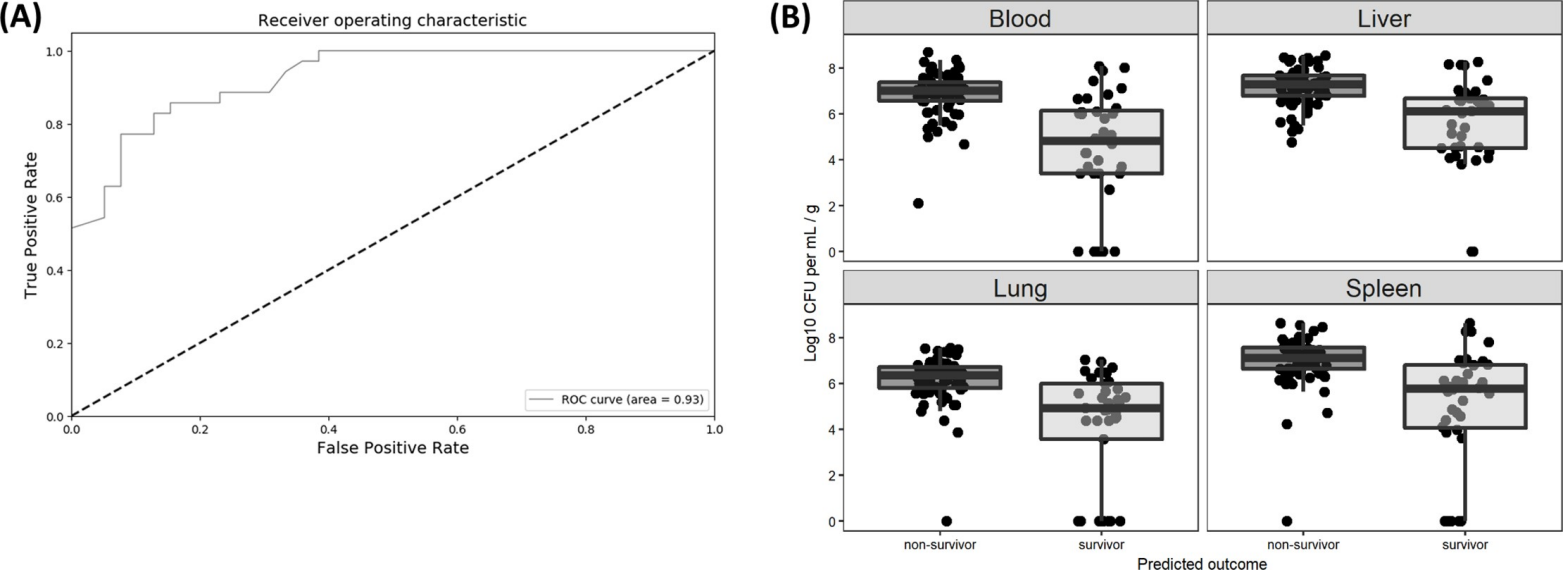
Survival or non-survival of neonatal mice was predicted at 24 HPC and was strongly associated with bacterial load. (A) Receiver Operating Characteristic curve illustrating the sensitivity and specificity of a Gradient Boosting Machine model trained on 148 mice and tested here on 74 mice. The model was primarily driven by weight change, righting reflex and mobility at 18 and 24 HPC. (B) Neonatal mice injected IP with cecal slurry were sacrificed 24 hours post challenge, with tissue and blood samples plated on sheep’s blood agar at time of sacrifice. Mice were predicted to survive or not survive based on righting reflex, mobility, and other biometric data. Mice which were predicted to survive had a significantly lower bacterial load in all measured biological compartments than predicted non-survivors (two-sided Wilcoxon rank-sum tests with Bonferroni correction, p < 0.001). All mice with zero bacterial load in the whole-blood (n = 9) were predicted to survive.

We used 10-fold cross validation training on a combination of six algorithms and three methods of feature selection in order to identify the most effective method of classification (S1 Table). A more detailed description of this approach is available in the supplemental section appendix 1 (S1 Appendix). For the final predictions, we used Gradient Boosting Machine as our classifier, combined with features selected by Pearson correlation coefficients. This approach had a cross validation average accuracy of 0.91 with a low standard deviation of 0.06, indicating little risk of over or underfitting (S3 Fig). With the hyperparameters selected for our Gradient Boosting model, we built a model on the training data subset with known outcome and applied it to 33% of the original data set not used during the training phase. With a total of 74 cases to classify as “survivor” or “non-survivor”, the Gradient Boosting model performed well with an accuracy score of 0.85 and an AUC of 0.93 (Fig 4A), with 33 true positive cases classified as “non-survivor” and 30 cases classified as “survivor” (Table 2). To further validate our model, we generated an additional dataset with the same data collection process as well as the same data cleaning and standardization pipeline. With a total of 21 new data points, our model accurately classified 18 with an average score of 85% accuracy (S2 Table). Righting reflex and mobility at 24 hours together with change in weight were the features most strongly correlated with outcome (S3 Fig). Finally, we applied our classifier to aa different set of pups sacrificed at 24 HPC and identified a clear relationship between survival and bacterial load (Fig 4B).

**Table 2:** Confusion matrix showing accuracy of Gradient Boosting Machine model when applied to test set of 74 mice. Using monitoring data collected at 18 and 24 HPC (weight, righting reflex and mobility), a Gradient Boosted machine learning model was able to distinguish survivors and non-survivors with an accuracy score of 0.85; 33/39 survivors and 30/35 non-survivors were correctly classified.

True class		
Survivor	**33**	6
Non-survivor	5	**30**
	Survivor	Non-Survivor
	**Predicted class**	

## Discussion

Here we present a system of health scores for neonatal mice which were used to confidently predict mortality 24 HPC in an established model of neonatal sepsis [19]. The significance of this is twofold: 1) the classifier enables investigators to include “clinical” outcome (e.g. mortality) as a covariate of interest when all animals are sacrificed, and 2) the scoring system can represent a new standard in recording and reporting neonatal mouse health which has implications beyond this specific model. Given the tremendous number of biological differences between neonates and adults which have been revealed in the last few decades [6,21–26], it is no longer tenable to assume data generated from adult animal studies would be relevant to neonatal infectious disease. In order to study neonatal diseases, it is critical to work with neonatal animals, though this presents a unique set of challenges which may act as a barrier towards investigators who may consider working with neonatal mice[27]. The inability to ethically collect a meaningful volume of blood (>2 μL) without sacrifice precludes investigators from pairing survival data directly with data generated from any biological sample. Thus, the significance of this work is three-fold: (1) investigators may use this classification method to include mortality as a covariate of interest when all animals are sacrificed, (2) the scoring system can represent a new standard in recording and reporting neonatal mouse health which can standardize neonatal mouse work across different laboratories, and (3) animal suffering for future work can be minimized with a uniform, quantitatively established humane endpoint.

We also demonstrated that survival can still be assigned even for neonatal mice sacrificed 24 HPC; without such an approach, one is forced to rely on biomarkers (i.e. inflammatory cytokines, cell mobilization, etc.) as a proxy outcome. The decision to separate scores into their base components (righting reflex / mobility on each side) was made to maximize the amount of information being input into the classifier. Where human interpretation of data requires simplicity, i.e. a single score, machine learning approaches have the capacity to parse much more complex data and identify meaningful relationships. Given that it is increasingly unclear whether death from sepsis is associated with an inflammatory or an anti-inflammatory response [3,6,21,28,29], it is critical to minimize reliance on biomarkers, as most lack rigorous evidence of their relevance to survival. Our choice of bacterial load as an outcome of interest served a dual purpose, it: 1) validated the legitimacy of the scoring system and classifier, as a correlation between outcome and bacterial load is often assumed [13,30], and 2) provided evidence that there truly is a link between bacterial load and outcome in this model of polymicrobial sepsis. Thus, already at this stage we were able to remove one assumption which underlies neonatal mouse work. While a link between outcome and bacterial load may seem unsurprising, this demonstrates a proof of concept for situations wherein one must rely on a less obvious biomarker as a proxy outcome (i.e. sterile inflammatory models, inactivated bacteria, etc.). These health scores and classification approach provide a direct mechanism for assigning survival and non-survival rather than solely observing inflammatory markers and assuming a relationship which may or may not exist [18].

We were surprised to see that righting reflex alone was strongly correlated with bacterial load across all measured biological compartments, indicating it was a robust metric of neonatal mouse health (Fig 1). The significance of righting reflex was also apparent in the process of feature selection during classifier construction–righting reflex was the single most correlated feature with survival (S4 Fig). Given the simplicity of collecting, these data suggest righting reflex should be recorded whenever neonatal mouse well-being is in question. Righting reflex in neonatal mice has previously been used as a tool to study behavioral development but has never been used to measure disease progression nor assess overall health in the context of an infection [31]. These data represent an important contribution towards describing neonatal mouse health which can help to inform ethical decisions around humane endpoints.

A potential limitation of this study is that mice which were non-mobile and failed to right on both sides (score 0) were sacrificed for ethical concerns, potentially violating the assumption of independence between score and survival. We addressed this by excluding mice who received a score of 0 from statistical analyses when survival was the outcome of interest. Further, the high bacterial load observed in whole-blood and the various organs independently validated the biological relevance of the scoring system presented. The use of righting reflex as a decision point in humane endpoint can help to standardize mouse models and reduce unnecessary suffering for newborn mice. In fact, the extremely low survival rate (<10%) of mice which failed to right and were lethargic 24 HPC suggests that this should be used as the humane endpoint for future studies. This approach could be further improved by rigorously selecting the times at which monitoring data were collected in order to maximize the potential difference between survivors and non-survivors whilst minimizing the potential cost of survivor bias. Finally, there are also some questions surrounding the use of righting reflex as a metric in younger mice which may be unable to right even at a healthy state–further research is warranted prior to assigning these health scores to mice prior to DOL 7.

The availability of a robust scoring system, with the additional proof that it can be predictive of outcome, should allow for a stratification of experimental groups beyond treated / untreated, or challenged / unchallenged, but also treated survivors / treated non-survivors, etc. The latter comparison will provide mechanistic insight into responders and non-responders of novel treatments–precisely what one hopes to attain from working with an animal model. Neonatal mouse models provide a mechanism to explore neonatal infectious disease in a manner that is unparalleled in humans. The ability to sacrifice and examine neonatal mice while not losing the potential associations with survival has the potential to break open the mysteries of neonatal sepsis. Here we demonstrated that bacterial load across all measured biological compartments was strongly correlated with our predictive health scores and therefore final outcome in neonatal polymicrobial sepsis. This study represents a blueprint for how to report neonatal mouse health in infectious disease models, and how to use that data to examine survival in mice which had to be sacrificed for sample collection.

## Supporting information

S1 Fig Mortality and decrease in score were not a result of damage associated with injection. One mouse per litter received a sham challenge of either PBS or dextrose 5% water and the scores were recorded at 18 and 24 HPC. (A) Mice which received sham challenges exhibited no mortality. (B) Mice which received sham challenges had significantly higher scores at 18 HPC than their littermates (two-sided Wilcoxon rank-sum tests with Bonferroni correction, p < 0.001). In this cohort, most mice have recovered by 24 HPC so there is no significant difference between the groups (p = 0.06) but the sham challenged were clearly healthier.
(TIF)

S2 Fig Distribution of scores assigned to neonatal mice at 18 and 24 hours post challenge. Scores assigned to neonatal mice 18 and 24 hours post IP challenge with cecal slurry (HPC). At 18 HPC the scores are poorly distributed, with the vast majority of mice assigned a score of 2 (failure to right, mobile) indicating that most mice have not progressed towards survival or non-survival. By 24 HPC the scores are evenly distributed as mice have begun to succumb to or recover from sepsis.
(TIF)

S3 Fig Feature selection visualization using Pearson correlation. Heatmap of feature correlations of with Pearson correlation. Scores were split into components, starting with looking at righting reflex and mobility independent from one another and then further separated by the lower and higher measurements of each score taken in duplicate. Change in righting reflex reflects the difference between the monitoring timepoints at 18 HPC and 24 HPC.
(TIFF)

S4 Fig Evaluation of baseline and ensemble algorithms in their ability to classify survivors and non-survivors in a neonatal mouse model of polymicrobial sepsis. Algorithms were trained on a set of 148 pups and tested on another set of 74, the accuracy (number of correct classifications over total number of classifications made) is shown on the y-axis.
(TIF)

S5 Fig 10-Fold cross validation of the Gradient Boosted Machine algorithm. (TIF)

S1 Table Accuracy of different algorithms and feature selection approaches in classifying mice into survivors or non-survivors at 24 HPC. (XLSX)

S2 Table Confusion matrix showing Gradient Boosting Machine algorithm applied to external dataset. Confusion matrix demonstrating the predictive capacity of the classifier when applied to a never seen dataset of 21 neonatal mice challenged with the cecal slurry model of sepsis. The classifier accurately categorized 9/11 survivors and 9/10 non-survivors, in line with the internal cross-validation set.
(XLSX)

S3 Table User-defined hyperparameters to be tuned for each algorithm. Hyperparameters must be set prior to initiating the training process and are critical to constructing a robust classifier.
(XLSX)

S1 URL Public GitHub repository containing the code used to construct the classifiers. https://github.com/radaniba/Sepsis_Project.
(DOCX)

S1 Appendix Classifier construction and background. More detailed outline of how the classifier was constructed, including background information providing context for technical decisions.
(DOCX)

S2 Appendix Reference guide for associated data. Explains the contents of each file associated with the manuscript.
(DOCX)
