# Supplementary figures and images for: Robust health-score based survival prediction for a neonatal mouse model of polymicrobial sepsis
Source: PLoS One. 2019 Jun 24;14(6):e0218714. doi: 10.1371/journal.pone.0218714 (PMC6590826; doi:10.1371/journal.pone.0218714)

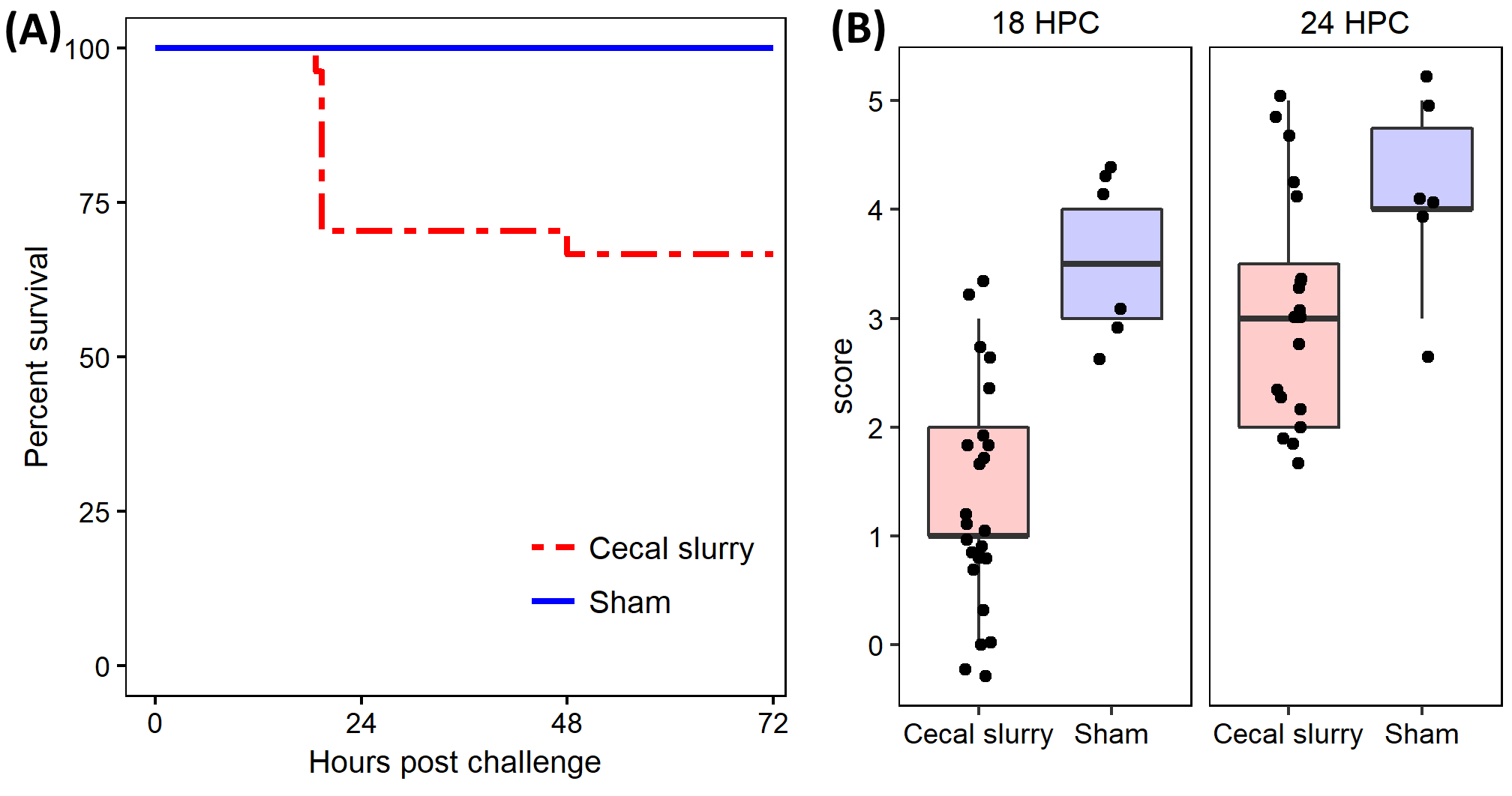

Supplement: S1 Fig — One mouse per litter received a sham challenge of either PBS or dextrose 5% water and the scores were recorded at 18 and 24 HPC. (A) Mice which received sham challenges exhibited no mortality. (B) Mice which received sham challenges had significantly higher scores at 18 HPC than their littermates (two-sided Wilcoxon rank-sum tests with Bonferroni correction, p < 0.001). In this cohort, most mice have recovered by 24 HPC so there is no significant difference between the groups (p = 0.06) but the sham challenged were clearly healthier. (TIF) [file pone.0218714.s001.tif]

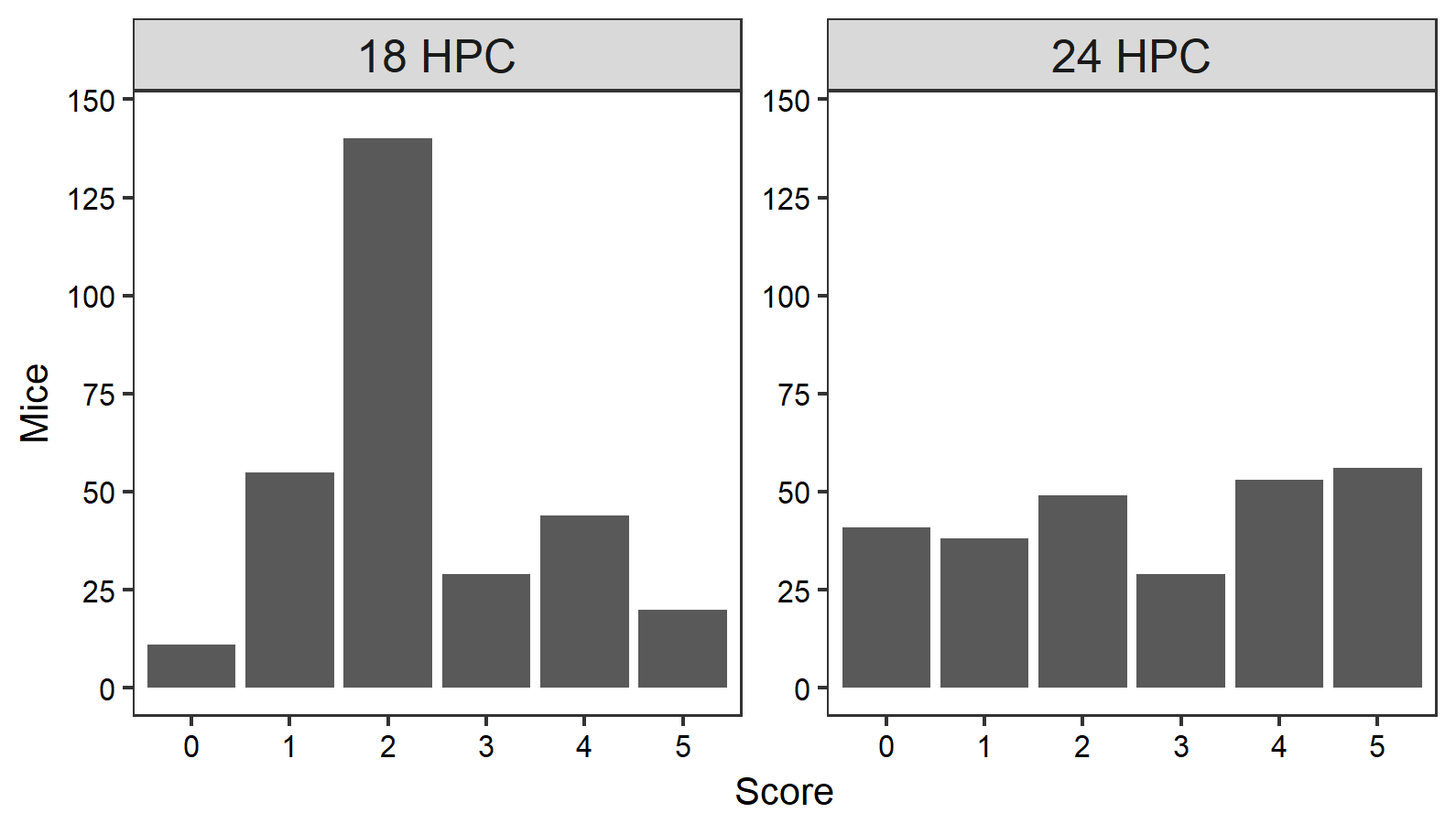

Supplement: S2 Fig — Scores assigned to neonatal mice 18 and 24 hours post IP challenge with cecal slurry (HPC). At 18 HPC the scores are poorly distributed, with the vast majority of mice assigned a score of 2 (failure to right, mobile) indicating that most mice have not progressed towards survival or non-survival. By 24 HPC the scores are evenly distributed as mice have begun to succumb to or recover from sepsis. (TIF) [file pone.0218714.s002.tif]

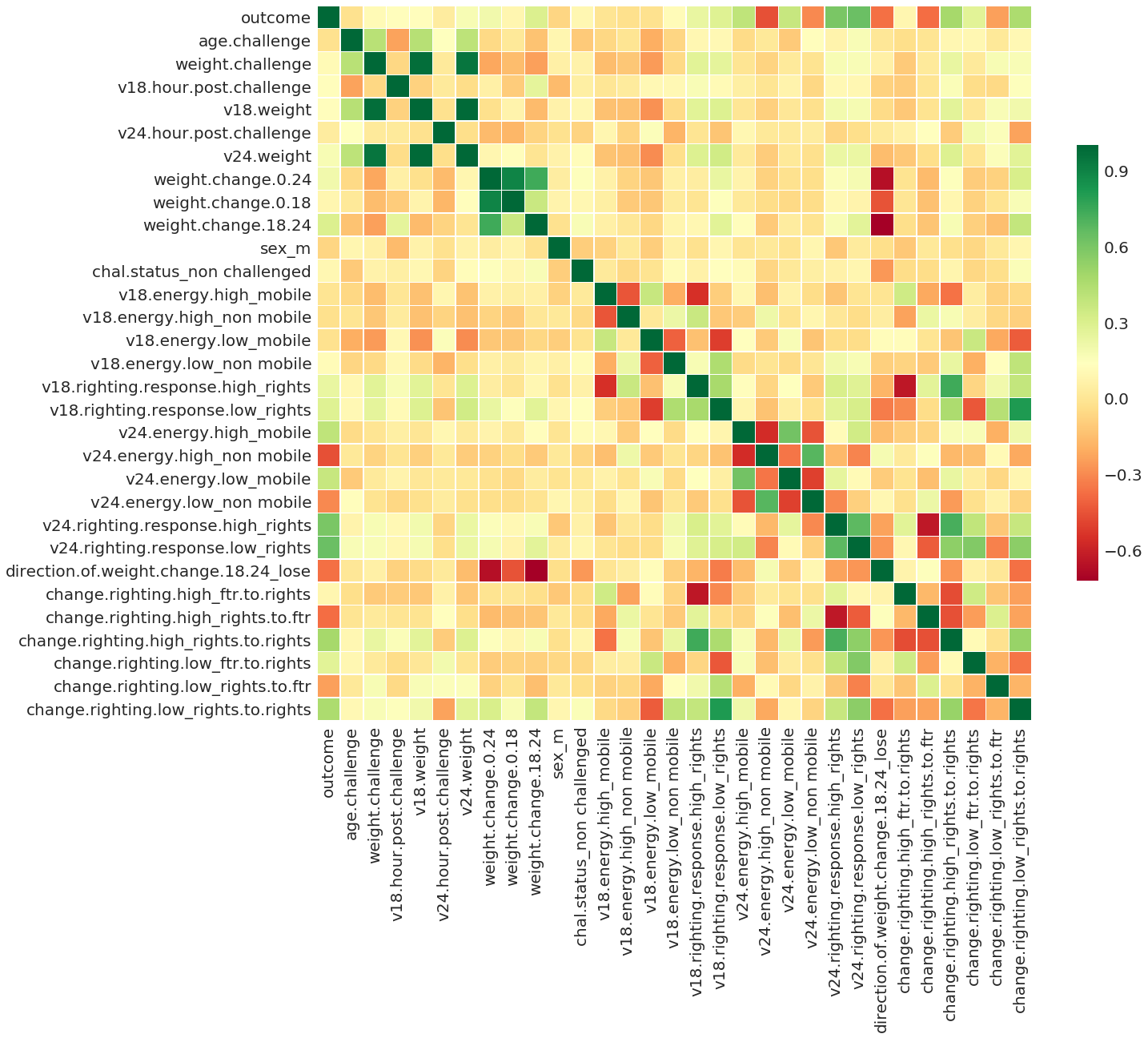

Supplement: S3 Fig — Heatmap of feature correlations of with Pearson correlation. Scores were split into components, starting with looking at righting reflex and mobility independent from one another and then further separated by the lower and higher measurements of each score taken in duplicate. Change in righting reflex reflects the difference between the monitoring timepoints at 18 HPC and 24 HPC. (TIFF) [file pone.0218714.s003.tiff]

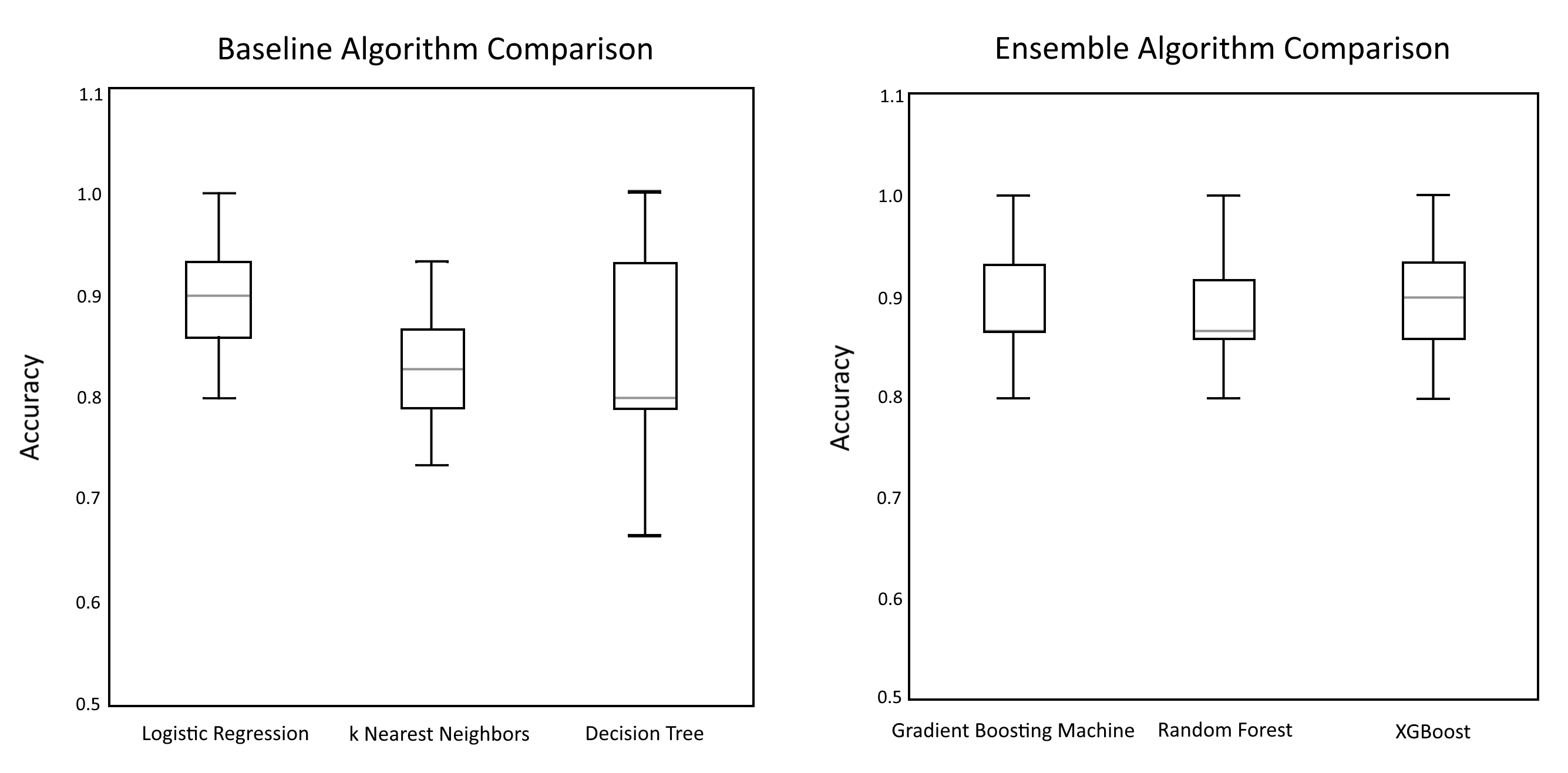

Supplement: S4 Fig — Algorithms were trained on a set of 148 pups and tested on another set of 74, the accuracy (number of correct classifications over total number of classifications made) is shown on the y-axis. (TIF) [file pone.0218714.s004.tif]

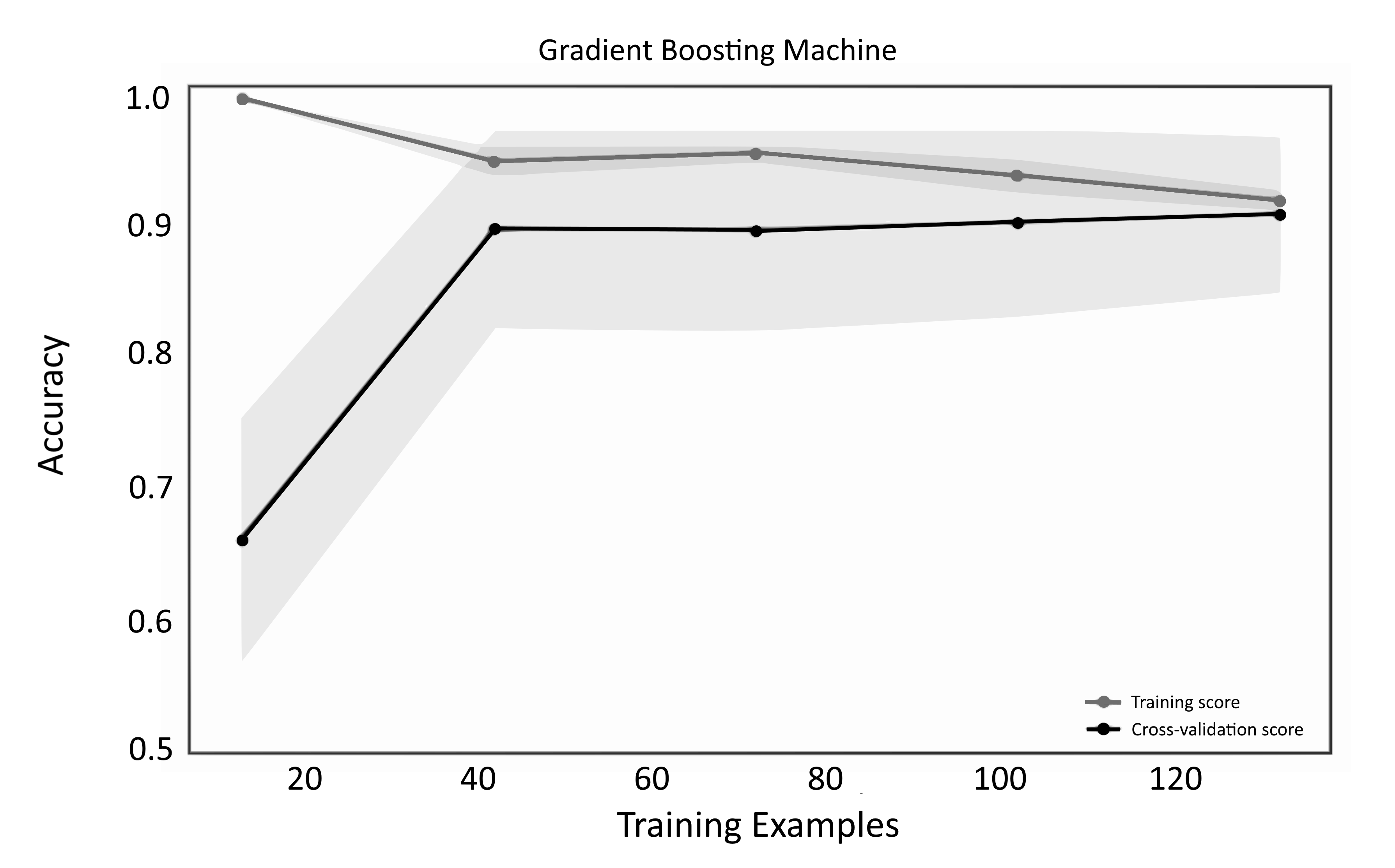

Supplement: S5 Fig — (TIF) [file pone.0218714.s005.tif]
