# Supplementary material for: Robust health-score based survival prediction for a neonatal mouse model of polymicrobial sepsis
Source: PLoS One. 2019 Jun 24;14(6):e0218714. doi: 10.1371/journal.pone.0218714 (PMC6590826; doi:10.1371/journal.pone.0218714)
Supplement: S1 URL — https://github.com/radaniba/Sepsis_Project. (DOCX) [file pone.0218714.s009.docx]

**Supporting information**

**S1 URL. Public GitHub repository containing the code used to construct the classifiers.**

<https://github.com/radaniba/Sepsis_Project>
