# Supplementary material for: Robust health-score based survival prediction for a neonatal mouse model of polymicrobial sepsis
Source: PLoS One. 2019 Jun 24;14(6):e0218714. doi: 10.1371/journal.pone.0218714 (PMC6590826; doi:10.1371/journal.pone.0218714)
Supplement: S1 Appendix — More detailed outline of how the classifier was constructed, including background information providing context for technical decisions. (DOCX) [file pone.0218714.s010.docx]

**Supporting information**

**S1 Appendix. Classifier construction and background**

Our goal was to construct a classifier which could predict whether mice would survive or die 24 hours post challenge (HPC) in a cecal slurry model of neonatal sepsis. The 24-hour time point was selected as it was the final monitoring point before many mice began to succumb to the infection, and thus represented the largest likely gap between the two groups of interest (survivors and non-survivors). As this dataset was assembled over more than a year, the first step was a cleaning process which consisted mainly of removing all data columns that were not relevant to the model training, as they were derived from experiment notebooks and were mainly used for tracking purposes. These attributes were mainly pup identifiers and dates of birth, litter identifiers *etc.* The remaining attributes were only those which could be potentially relevant to the prediction of outcome – this was the starting point for feature selection in our model building. The dataset was a mix of continuous (i.e. weight, hours post challenge) and categorical (i.e. sex, righting reflex) features. Categorical features were encoded as binary operators (1 or 0) using the dummy variable function in the Pandas package – this is required for some of the approaches we pursued.

The selection of the most relevant features to this classification problem is an important step and can be the difference between success and failure. Inclusion of irrelevant features may inject noise into the system and can reduce the overall accuracy. Here we deployed three approaches to feature selection: correlation-based selection, hypothesis testing selection, and machine learning selection. Briefly, correlation-based approaches compare correlation coefficients to identify those which are linearly dependent with one another and are therefore not necessary to include moving forward. Hypothesis testing is a more rigorous approach which statistically tests each feature and returns p-values indicating the likelihood that a given feature is relevant to the outcome of interest, in this case survival. Machine-learning selection relies on a supervised machine-learning algorithm (in this case we used Support Vector Classification) to rank the relevance of each feature to the desired outcome.

**Algorithms**

As the working dataset of 222 pups was small by data science standards, we were able to test multiple approaches without concern for time or computing power. Baseline algorithms are advantages in their ability to be understood and explained, whereas ensemble learning approaches act more as a ‘black box’ with the line from input to output becoming very difficult to follow. Of course, ensemble learning approaches generally have a higher capacity to model complex problems. Thus, in order to get a sense of which approach was most appropriate for this specific classification problem, we opted to test three baseline learning algorithms and three advanced ensemble learning algorithms.

We selected logistic regression, K Nearest Neighbors (kNN), and simple decision trees for our three baseline learning approaches. Logistic regression has the distinct advantage of being extremely simple to interpret – the output is a probability so it can be used not only in capacity to predict but also to rank confidence of each prediction. However, it is strongly susceptible to overfitting due to multicollinearity (feature correlation with one another) which was clearly present in this dataset (i.e. weight at 0 HPC is strongly related to weight at 18 HPC and weight at 24 HPC). kNN operates under the principal that similar treatments on different subjects will lead to similar outcomes; the principal advantage of kNN is that it requires no assumptions about the data and is non-parametric in its approach. The only information which must be user specified is the proper estimation of ‘k’. It is, however, highly sensitive to outliers and features in the dataset which may be irrelevant. kNN technically does not “learn” in the classic sense, rather it stores data and examines the similarity or proximity of new instances against those already stored. The final baseline approach we explored were simple decision trees, which are generally simple to interpret but even more so when the classification problem is binary in nature. Contrary to logistic regression and kNN, decision trees are insensitive to the linear separability of the data and thus there is less concern regarding outliers and multicollinearity. On the downside, decision trees are highly sensitive to small changes in user input data which can lead to large changes in the trees. Exposure to unexpected data is generally not well handled and can lead to errors in classification.

The more advanced, ensemble learning techniques we used were Random Forest, Gradient Boosting, and XGBoost algorithms. These ensemble techniques merge several weak learners into a single strong one, reducing bias and variance considerably in supervised learning use cases. The specific benefits and downsides of each of these algorithms is beyond the scope of this work. They are generally fast, user-friendly, and well-capable of handling mixed data types (categorial and continuous). These strengths though also make them prone to overfitting and noise, which is why it is extremely important to cross-validate with rigor. Each of these algorithms comes with a set of user-defined hyperparameters which must be set prior to beginning the training, and optimizing these parameters is a critical step in attaining accurate classifications – the parameters are listed in S3 Table. We optimized these parameters using the GridSearch algorithm, which tests all possible combinations of values and returns the most effective values for a given model. For precise parameters used in each case, refer to accompanying code linked in the supplementary materials. The strength of each algorithm was assessed internally using 10-fold cross validation wherein the model is trained and tested iteratively on different subsets of the data. The strongest approach (Gradient-Boosted machine learning) was then tested on an external subset of data which had not been seen by the model.

**Feature selection**

As feature selection is a widely discussed subject in machine learning, we have different ways of selecting a subset of features that are the most relevant to our classification problem. Using irrelevant features might make the learning process longer and more expensive in terms of resources and could also make the final prediction not accurate and biased because of noise added to the model training.

**Correlation based feature selection**

In this paper we used Pearson correlation to compare the features. Highly correlated features were more linearly dependent and hence had almost the same effect on the dependent variable (in our case, the outcome being the life or death of the mouse): we then might drop one of the correlated features as there will be no effect on the final outcome.

**Hypothesis testing feature selection**

We used hypothesis testing as a more complex statistical approach to select the best features from our dataset using a more rigorous feature choice by looking at the p-values. The main purpose of choosing this approach was to examine whether or not certain conditions could be applied to an entire population, from a data sample. The result of a hypothesis test determines if we should believe the hypothesis or reject it for an alternative one. To select the best features in our dataset we were interested in testing the hypothesis of a given feature having no relevance to the response variable. We wanted to test this hypothesis for every feature and decide whether the features hold some significance in the prediction of the response. Based on the p-value per feature, the general rule was that the lower the p-value, the better the chance that we can reject the null hypothesis. In our case, the smaller the p-value, the better the chances that the feature had some relevance to outcome and it should be retained moving forward.

**Feature selection using a machine learning algorithm**

We investigated the possibility of using a machine learning algorithm as a proxy for selecting a subset of features for the rest of the analysis. We used Support Vector Classifier as a method generally recommended to perform for ordinal classification (taking in consideration that outcome was transformed into binary values). Because we are using a machine learning model to rank features, we have an additional complication which consists in the fact that we have an additional hyperparameters to optimize. To tackle this issue, we use the same principle with GridSearch algorithm and combining the feature selection and the training phase optimization within the same pipeline (see details in the notebook attached)

**Choosing the best model**

In order to validate the use of righting reflex and mobility as metrics of health scores in neonatal mice, we used the machine learning approach described above to classify mice as survivors or non-survivors based primarily on righting reflex, mobility, and weight, specifically looking at how each metric changed from 18 to 24 HPC. Feature selection and classification was performed on components of the scores (righting reflex and mobility) rather than directly on the scores themselves in order to independently quantify the significance of each metric, in comparison to other commonly collected biometric data (i.e. weight). Only 12% (28 / 222) of mice were at humane endpoint at 24 HPC, so while the association between score components and outcome may be influenced by the inclusion of these mice (mice which were FTR non-mobile on both sides will, by definition, not survive) they did not represent a large enough population to call into question the results.

Some of the features describing the dataset were categorical. Because some algorithms are known to be dealing with this kind of feature types better than other (like decision trees for example), we decided to encode these categorical features into numerical ones, using the one-hot encoding technique, thus creating a numerical fingerprint per training data point. For instance, the feature ‘change_righting_high’ describing (….) has categorical values such as ‘ftr.to.rights’ or ‘rights.to.ftr’. When the encoding is applied this feature is then transformed into two newly engineered ones ‘change_righting_high.ftr.to.rights’ and ‘change_righting_high.rights.to.ftr’ with binary values (0, 1). Following this feature engineering process and data cleaning and standardization, we examined the correlation between these attributes and the final outcome and decided to select the features having a correlation with an absolute value higher than 0.2 as a threshold. The heatmap shown in S3 Fig is a graphical representation of Pearson correlation applied to the feature matrix.

Except few attributes highly correlated, we can observe a certain attributes independence, which could be beneficial for the final accuracy of the trained model. Highly collinear features may be a bias in the training phase, as they describe the data point the same way and bring no variability to learn different patterns which may cause either overfitting or underfitting the model to the training set. In order to have the optimal model, we need to account for two parts. First, we need to make sure that we are using the attributes that describe the best our data patterns, and second, we need to use the best algorithm with these selected attributes. Thus, we have a combination of six algorithms and three feature selection methods. To add some complexity on the mix, every algorithm comes with different parameters that we need to play with to tune the model, luckily, we have the Grid Search method which is a hyperparameter optimization process that will test all possible combination of values and give us the best values to use for the model. We performed a 10-fold cross validation training using all these items described above and we measured the training accuracy for each use case. The result is displayed in S1 Table.

We can clearly see that, for our small dataset, the accuracy is high for all the algorithms used (> 0.8) which is a very good indicator that the problem we tried to solve (predicting mortality) is predictable given the data set in hand. We need to be sure about the optimal solution though by minimizing the error rate as much as possible in order to inform accurately on the subject we need to use the biological material from subsequent experiments. Gradient Boosting and XGBoost are two algorithms that, combined with feature selection based on correlation, gave the best training accuracy compared to the other methods (S1 Table). We thus decided to select Pearson correlation and these two ensemble learning to build our optimal solution. S4 Fig shows the training accuracy per Classifier family. We decided to use the Gradient Boosting Machine as our classifier combined with features selected by Pearson correlation. The S5 Fig below describes how this accuracy is reached.

**Testing the Gradient Boosting Machine model**

With the hyperparameters selected for our Gradient Boosting model, we built a model on the training dataset and applied it to a 33% of the original data set not used during the training phase. The goal is to test whether or not our model is generalizable. With a total of 74 cases to classify as “Live” or “Die”, our model using Gradient Boosting performed extremely well with an accuracy score of **0.85** and an ROC value of **0.93**, with 33 True Positive cases classified as “Die” and 30 cases classified as “Live”, the confusion matrix shown in S2 Table shows the accuracy of our model. To further validate our model, our team generated an additional dataset with the same data collection process as well as the same data cleaning and standardization pipeline. With a total of 21 new data points, our model was able to accurately classify 18 with an average score of **85% accuracy** (S2 Table).
