# Supplementary material for: Robust health-score based survival prediction for a neonatal mouse model of polymicrobial sepsis
Source: PLoS One. 2019 Jun 24;14(6):e0218714. doi: 10.1371/journal.pone.0218714 (PMC6590826; doi:10.1371/journal.pone.0218714)
Supplement: S2 Appendix — Explains the contents of each file associated with the manuscript. (DOCX) [file pone.0218714.s011.docx]

**Supporting information**

**S2 Appendix. Reference guide for associated data**

**Data file overview**

**mouse_mortality_data.xlsx [266 mice]**

This is the data set used for all the explorations into score, righting reflex and mortality. All the mice from classifier_building_data.xlsx and classifier_validation_cohort.xlsx (combined 243 mice) are represented here. The additional 23 mice were excluded from model construction due to incomplete data collection (i.e. missing weight, monitored at 21 hours and 30 hours rather than 24 hours, etc.) but were still able to be used in things such as survival curves.

**classifier_building_data.xlsx [222 mice: subset of mouse_mortality_data.xlsx]**

This was the data used to train and internally test the model. It is presented separately as these were the features that were presented as the starting point for classifier construction. For internal cross-validation, this set was further split into a training set (66%, 148 mice) and test set (33%, 74 mice).

**classifier_validation_cohort.xlsx [21 mice: subset of mouse_mortality_data.xlsx]**

This was the 21-mouse external validation cohort used to generate Figure S3 and validate the classifier. As above, it is a subset of mouse_mortality_data.xlsx filtered to only include features which were presented to the classifier.

**mouse_CFU_data.xlsx [125 mice]**

These mice were all sacrificed at 24 HPC, dissected to calculate bacterial load in blood, spleen, liver, lung, and then classified into predicted survivors or predicted non-survivors.

**sham_challenge_control_set.xlsx [33 mice]**

These mice were included exclusively to show the morbidity and change in score was not associated with the needle poke. Used only to generate supplementary figure 1 (S1 Fig).
